# Supplementary material for: A simple immunoassay for extracellular vesicle liquid biopsy in microliters of non-processed plasma
Source: J Nanobiotechnology. 2022 Feb 8;20:72. doi: 10.1186/s12951-022-01256-5 (PMC8822649; doi:10.1186/s12951-022-01256-5)
Supplement: Supplementary file 1 — Additional file 1: Table S1. Lung cancer patients. Demographic and pathological data. Figure S1. Bead-assisted immunocapture does not result in binding of all the vesicles available in the mixture. 6000 antiCD63-coated beads were incubated with 2 µg of PC3-derived EVs and captured vesicles were detected by flow cytometry after incubation with anti-CD9-PE. IgG was used as a negative control. Supernatants of the first incubation (SN 24h), containing unbound exosomes, were incubated again with anti-CD63 beads and EVs captured during this second incubation were analysed by flow cytometry. This procedure was repeated the following 2 days (SN 48 h, SN 72 h). A. Histograms from a representative experiment out of 4. B. Relative Fluorescence Intensity (RFI) values. Figure S2. Polydispersity Index in the diameter measured by DLS increase in the presence of charged polymers. Metastatic melanoma (Ma-Mel-86c) derived EVs, obtained by ultracentrifugation, were diluted in HBS and incubated during 18 h with or without 8 μg/ml Polybrene (PB) or 4 μg/ml Poly-L-lysine (PL). The hydrodynamic diameter was measured by Dynamic Ligh Scattering (DLS). Intensity Mean (average diameter in nm) data from one representative experiment is shown together with the associated Polydispersity Index (PdI). Figure S3. Concentration and Z-potential measurement using NTA (Zetaview Technology). Ma-Mel-86c Metastatic melanoma-derived EVs obtained by ultracentrifugation were diluted 1:64000 in PBS and incubated during 5 minutes with either 8 μg/ml Polybrene (PB) or 1 μg/ml Poly-L-lysine (PL). Concentration and Z- potential were measured by NTA using a Zetaview Technology instrument. Measurement Parameters: Cell S/N: ZNTA-405. Sensed Electric Field: 3.1 V/cm (pulsed). Measurement Mode: Profile 11 Positions. Sensed Temperature: 31 ºC. The addition of cationic polymers led to an increase in the value of Z-potential. Figure S4. Sedimentation coefficient profile of EVs in the presence of charged polymers. Metasta [file 12951_2022_1256_MOESM1_ESM.pdf]

A Simple Immunoassay for Extracellular Vesicle Liquid Biopsy in Microliters of Non-Processed Plasma

Carmen Campos-Silva[1], Yaiza Cáceres-Martell[1], Estela Sánchez-Herrero[2, 3], Amaia Sandúa [4], Alexandra Beneitez-Martínez[5], Álvaro González [4], Mariano Provencio[2,6], Atocha Romero[2, 6], Ricardo Jara-Acevedo[5], María Yáñez-Mó[7, 8] and Mar Valés-Gómez[1#]

SUPPLEMENTARY FIGURES AND TABLE

[1] Department of Immunology and Oncology, Spanish National Centre for Biotechnology, CNB-CSIC, Madrid, Spain

[2] Laboratorio de Biopsia Líquida. Instituto de Investigación Sanitaria Hospital Universitario Puerta de Hierro, Majadahonda, Madrid, Spain

[3] Atrys Health, Barcelona, Spain.

[4] Service of Biochemistry, Hospital Universitario Clínica Universidad de Navarra, Pamplona, Spain

[5] Immunostep, S.L., Salamanca, Spain

[6] Medical Oncology Department. Hospital Universitario Puerta de Hierro. Majadahonda, Madrid, Spain

[7] Department of Molecular Biology, UAM - Centro de Biología Molecular Severo Ochoa, Madrid, Spain

[8] Instituto de Investigación del Hospital Universitario La Princesa, Madrid, Spain

Table S1. Lung cancer patients. Demographic and pathological data

Characteristics		Initial stage lung cancer (N=12)	Advanced stage lung cancer (N=12)	Healthy donors (N=12)
		Number (%)	Number (%)	Number (%)
Age	Mean	68.83	66.08	38.66
	Min-Max	51-87	45-78	22-56
Sex	Male	8 (66)	7 (58.33)	3 (25)
	Female	4 (33)	5 (41.66)	9 (75)
Histology	**Non-Small Cell Lung Cancer (NSCLC)**	9 (75)	9 (75)	
	- Adenocarcinoma	- 8 (66.66)	- 8 (66.66)	
	- Squamous cell carcinoma	- 1 (8.33)	- 1 (8.33)	
	- Large cell carcinoma	- 0 (0)	- 0 (0)	
	**Small cell lung cancer (SCLC)**	1 (8.33)	2 (16.66)	
	**Other (pleural mesothelioma or epidermoid carcinoma)**	2 (16)	1 (8.33)	
Clinical Stage	IA	3 (25)		
	IB	2 (16.66)		
	IIA	1 (8.33)		
	IIB	6 (50)		
	IIIA		2 (16.66)	
	IIIB		2 (16.66)	
	IV		8 (66.66)	

Fig. S1

A.

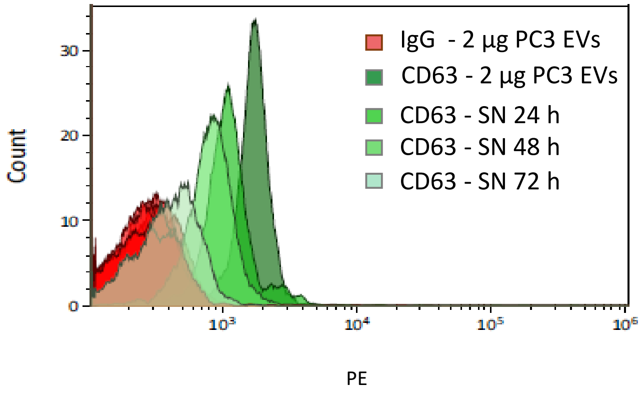

B.

Sample	RFI
CD63 – 2 µg PC3 EVs	6.27
CD63 - SN 24 h	4.55
CD63 - SN 48 h	3.69
CD63 - SN 72 h	1.68

Bead-assisted immunocapture does not result in binding of all the vesicles available in the mixture. 6000 anti-CD63-coated beads were incubated with 2 µg of PC3-derived EVs and captured vesicles were detected by flow cytometry after incubation with anti-CD9-PE. IgG was used as a negative control. Supernatants of the first incubation (SN 24h), containing unbound exosomes, were incubated again with anti-CD63 beads and EVs captured during this second incubation were analysed by flow cytometry. This procedure was repeated the following 2 days (SN 48 h, SN 72 h). **A.** Histograms from a representative experiment out of 4. **B.** Relative Fluorescence Intensity (RFI) values.

Fig. S2

Sample Name	Intensity Mean (diameter nm)	PdI
Untreated	293,7	0,285
Polybrene 8 ug/ml	444,6	0,712
Poly-L-lysine 4 ug/ml	874,5	1

Polydispersity Index in the diameter measured by DLS increase in the presence of charged polymers. Metastatic melanoma (Ma-Mel-86c) derived EVs, obtained by ultracentrifugation, were diluted in HBS and incubated during 18 h with or without 8 µg/ml Polybrene (PB) or 4 µg/ml Poly-L-lysine (PL). The hydrodynamic diameter was measured by Dynamic Ligh Scattering (DLS). Intensity Mean (average diameter in nm) data from one representative experiment is shown together with the associated Polydispersity Index (PdI).

SAMPLE	Original Concentration (particles/ml)	Z potential (mV)
Untreated	2.2E+12	-9.50 ± 0.40
8 µg/ml Polybrene	2.5E+12	9.27 ± 0.41
1 µg/ml Poly-L-lysine	4.1E+12	10.04 ± 0.05

Concentration and Z-potential measurement using NTA (Zetaview Technology). Ma-Mel-86c Metastatic melanoma-derived EVs obtained by ultracentrifugation were diluted 1:64000 in PBS and incubated during 5 minutes with either 8 µg/ml Polybrene (PB) or 1 µg/ml Poly-L-lysine (PL). Concentration and Z- potential were measured by NTA using a Zetaview Technology instrument. Measurement Parameters: Cell S/N: ZNTA-405. Sensed Electric Field: 3.1 V/cm (pulsed). Measurement Mode: Profile 11 Positions. Sensed Temperature: 31 ºC. The addition of cationic polymers led to an increase in the value of Z-potential.

Fig. S4

EVs isolated by ultracentrifugation

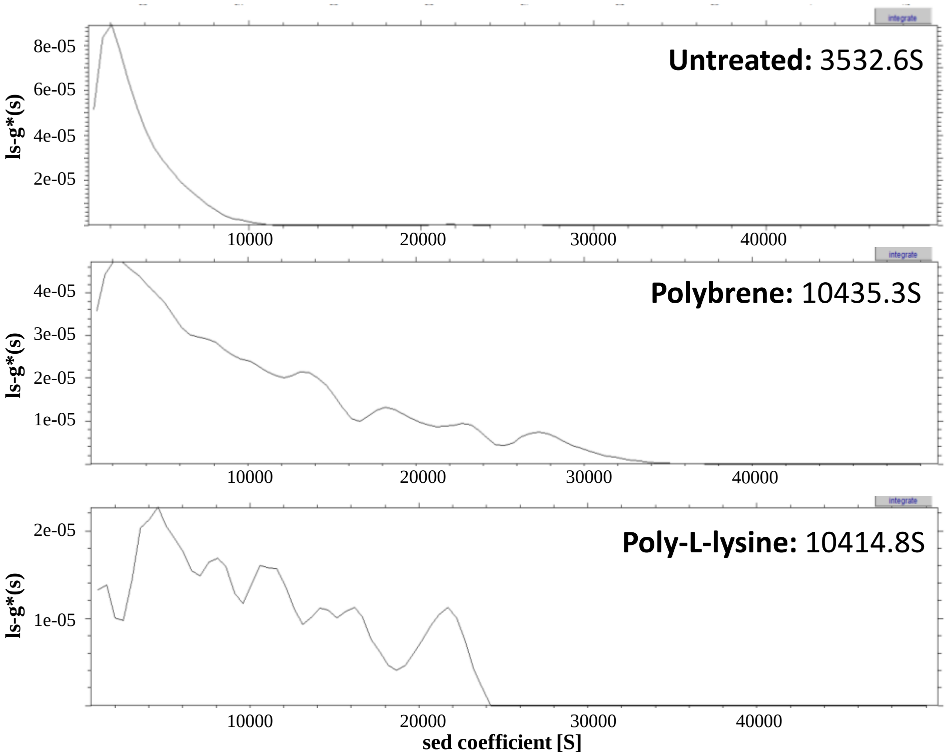

EVs isolated by SEC

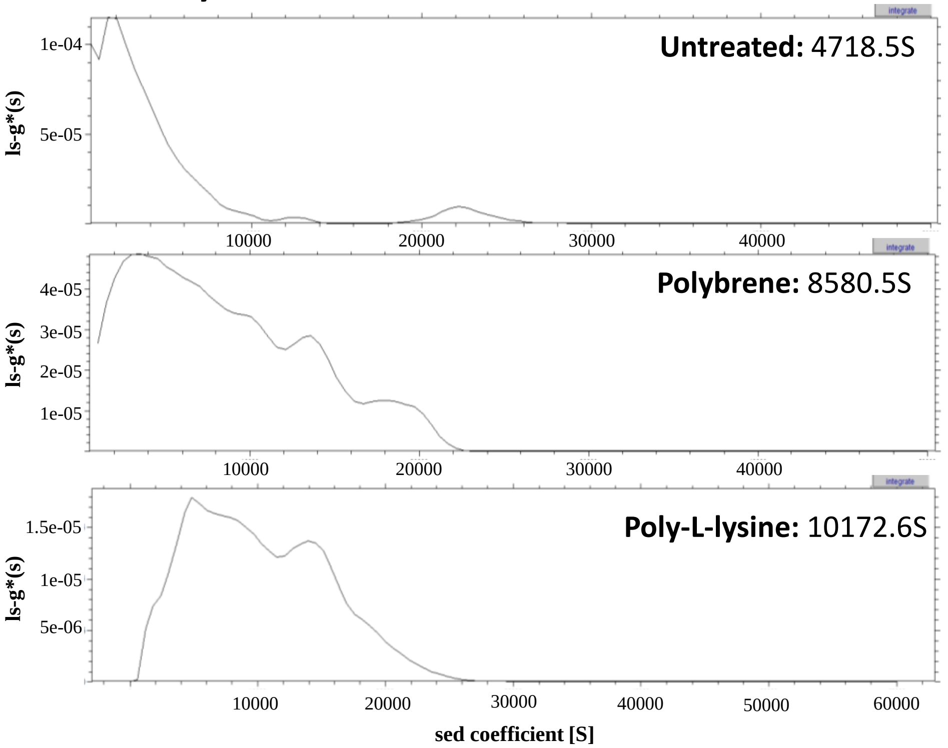

Sedimentation coefficient profile of EVs in the presence of charged polymers.

Metastatic melanoma (Ma-Mel-86c)-derived EVs obtained by ultracentrifugation or further isolated by SEC, as indicated, were diluted in HBS and incubated during 18 h with or without 8 µg/ml Polybrene (PB) or 4 µg/ml Poly-L-lysine (PL). Sedimentation coefficient profiles and weight (signal) average sedimentation coefficient (inset) were obtained by analytical ultracentrifugation. Polydispersity increased when charged polymers were added to the EV solution.

Fig. S5

A. NTA

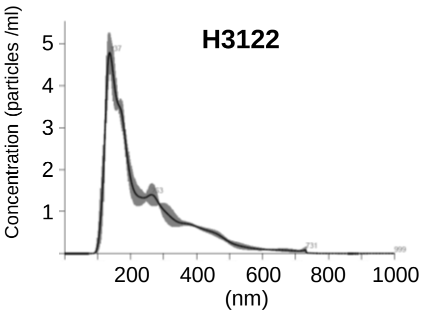

Average ϕ	244.2 +/- 6.1 nm
Mode ϕ	139.2 +/- 3.1 nm
Particles/µL	4.19 · 10^8

B. TEM

H3122

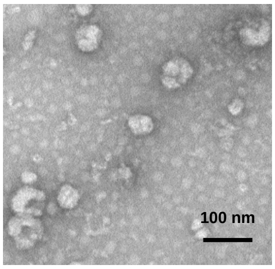

C. WB

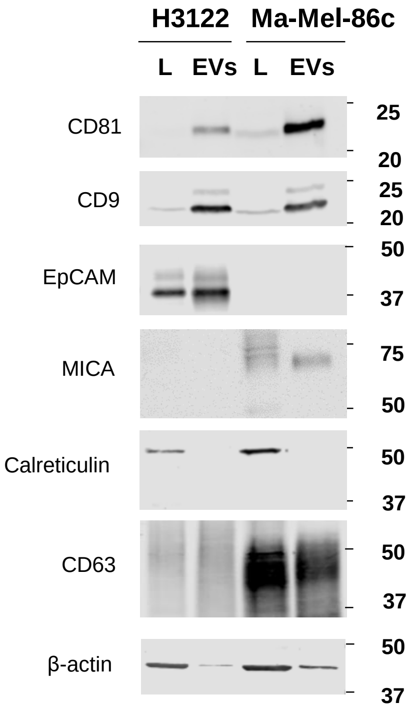

Characterization of cell lines-derived EVs. A. Size and concentration analysis by Nanoparticle tracking analysis (NTA). Average size and concentration listed in the table were obtained in a Nanosight equipment capturing 3 videos of 60 s per measurement, with camera level 12, threshold 10 and temperature of 25 °C. Software NTA 3.1 (Malvern) was used for the analysis. ϕ: diameter. **B. Transmission Electron Microscopy (TEM) visualization.** 1 µL EVs were diluted 1:10 in HBS and floated on a carbon-coated 400-mesh 240 Formvar grid, then incubated with 2% uranyl acetate and analysed using a Jeol JEM 1011 electron microscope operating at 245 100 kV with a CCD camera Gatan Erlangshen ES1000W. Pictures were taken at the Electron Microscopy Facility of the CNB. Bar: 100 nm. A representative image is shown. **C. Protein marker characterization by Western Blot.** EVs and whole cell lysates (L) were loaded in 12% SDS-PAGE gels. Membranes were immunoblotted for detection of: tetraspanins CD9, CD63, CD81 as general EV markers; β-actin as loading control; EpCAM and MICA as cancer-related markers; and calreticulin (CALR) as an endoplasmic reticulum resident protein not present in the EV fraction. Two gels were loaded: one gel, under non-reducing conditions and the other under reducing conditions, for actin detection. One representative experiment out of 3 is shown.

A. Flow Cytometry. Polymer titration in Ma-Mel-86c derived EVs

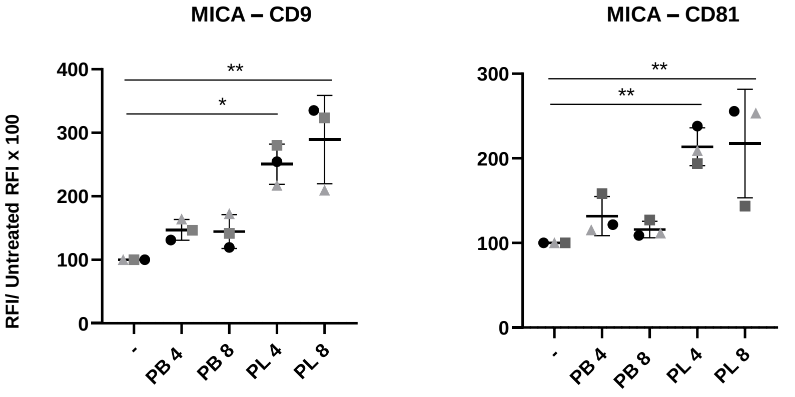

B. ELISA

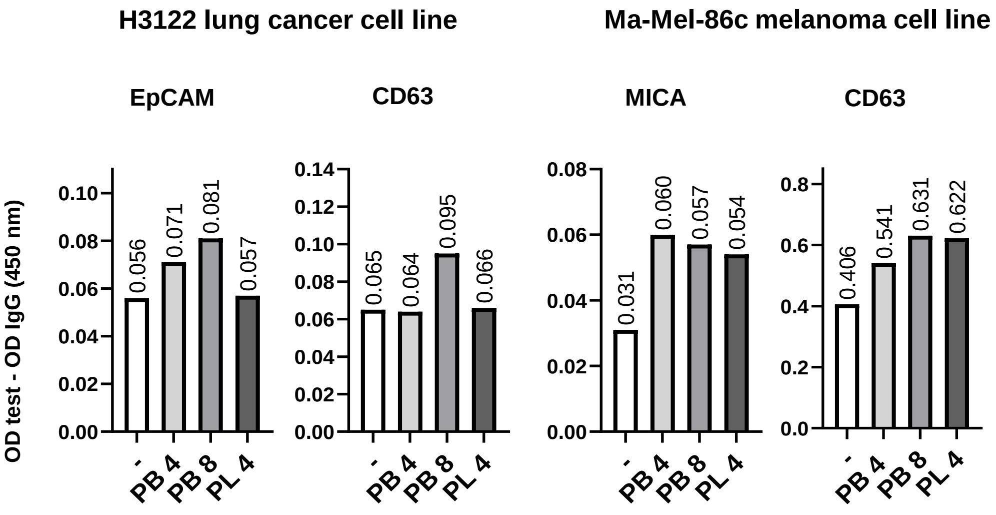

Cationic polymer addition increased cell lines-derived EV detection by Flow Cytometry and ELISA. A. Titration of cationic polymers. Tissue culture supernatant-derived EVs from the melanoma cell line H3122 were incubated with 3000 anti-MICA coated beads in 100 µl final volume of PBS-Casein 1%. MICA-captured vesicles were detected by flow cytometry after incubation with anti-CD9-PE anti-CD81-PE or isotype control. Relative increase of the RFI, obtained in three experiment replicates, is shown. Statistical analysis was performed by a Two-way ANOVA Fisher's LSD test. (* $p < 0.05$). **B. ELISA.** 100 µl containing 1 x 10^6 H3122 EVs/µl or 1.8 x 10^7 Ma-Mel-86c EVs/µl in PBS-Casein 1% were treated with 4 or 8 µg/ml polybrene (PB), 4 µg/ml poly-L-lysine (PL) or kept untreated and incubated for 18 h in anti-EpCAM, anti-MICA or anti-CD63 antibody-coated plates. IgG coated wells were used as isotype control. EV detection was performed after incubation with biotinylated anti-CD9 antibody followed by SA-HRP. Optical Density (OD) was measured at 450 nm. Optical Density (OD) of the samples was represented after substraction of isotype OD. A representative experiment out of 3 is shown.

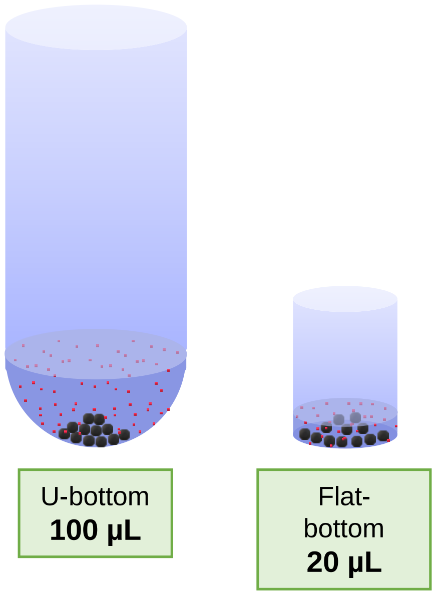

Volume reduction increased cell lines-derived EV detection. A. Schematic representation of the binding surface. Diameter dimensions of the cytometry tube and the well of a 96-well plate are depited to scale. Beads are represented in black and EVs in red.

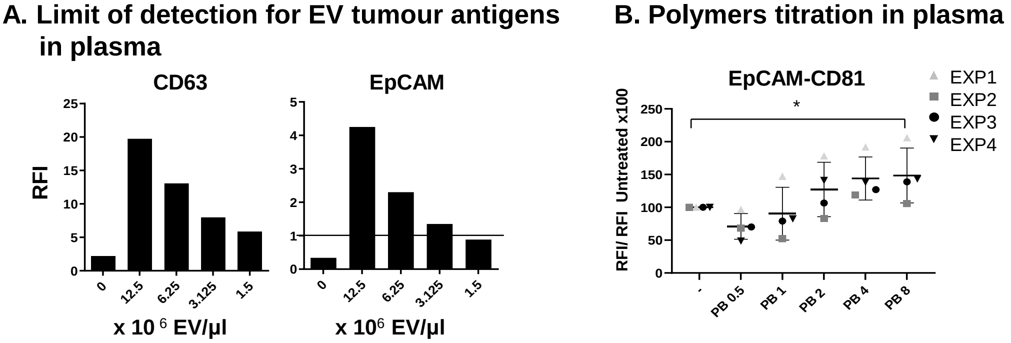

A. Limit of detection of tetraspanins and EpCAM in plasma EVs, after addition of lung cancer-derived EVs. 12 µl of plasma samples containing decreasing concentrations of lung cancer-derived EVs were incubated with 12 µl of PBS-casein containing 3000 anti-CD63, anti-EpCAM beads or IgG isotype coated beads. Captured EVs were detected with anti-CD81-PE. Bar plots represent RFI (Relative Fluorescence Intensity) values obtained. The limit of detection for EpCAM was below 3.125 x 10^{6} EVs/µl. **B. Titration of polymers for EpCAM detection in plasma after addition of lung cells-derived EVs .** 3000 anti-EpCAM or IgG isotype-coated beads were incubated for 18 h with 2.6 x 10^{6} H3122-derived EVs/µl in 12 µL of healthy donor's plasma in a final volume of 30 µL/test. Five different concentrations (0.5, 1, 2, 4 and 8 µg/ml) of Polybrene (PB) were compared to a polymer-untreated sample. Captured vesicles were detected by flow cytometry after incubation with anti-CD81-PE. IgG was used as a negative control. Increase of EpCAM-CD81 RFI relative to the untreated condition in four experiment replicates (EXP) is shown. Statistical analysis was performed by a Two-way ANOVA Fisher's LSD test. (Confidence Interval CI = 90%) (* $p < 0.1$). Healthy donor samples were obtained at the University Hospital Puerta de Hierro.

Fig. S9

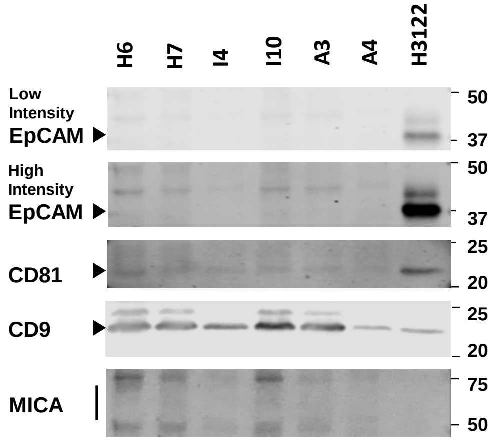

Western Blot detection of different proteins in the EV preparation by ultracentrifugation from lung cancer patient's plasma. 200 µl of plasma from 2 initial stage lung cancer patients (with EpCAM and MICA positive EVs detected by bead assisted flow cytometry), and from 2 healthy donors and 2 advanced stage patients plasma, were ultracentrifuged and the EV enriched preparation was resuspended in 15 µl. 5 µl of this EV preparation were loaded in a 12% SDS-PAGE gel and transferred to nitrocellulose. Lung cancer derived H3122 EVs (positive for EpCAM) were used as a positive control for EpCAM detection. The membrane was immunoblotted for detection of EpCAM and MICA and the tetraspanins CD9 and CD81, as general EV markers. EpCAM could not be detected even at high intensity exposure while MICA only showed a faint band in one of the initial stage patients after long exposure times.

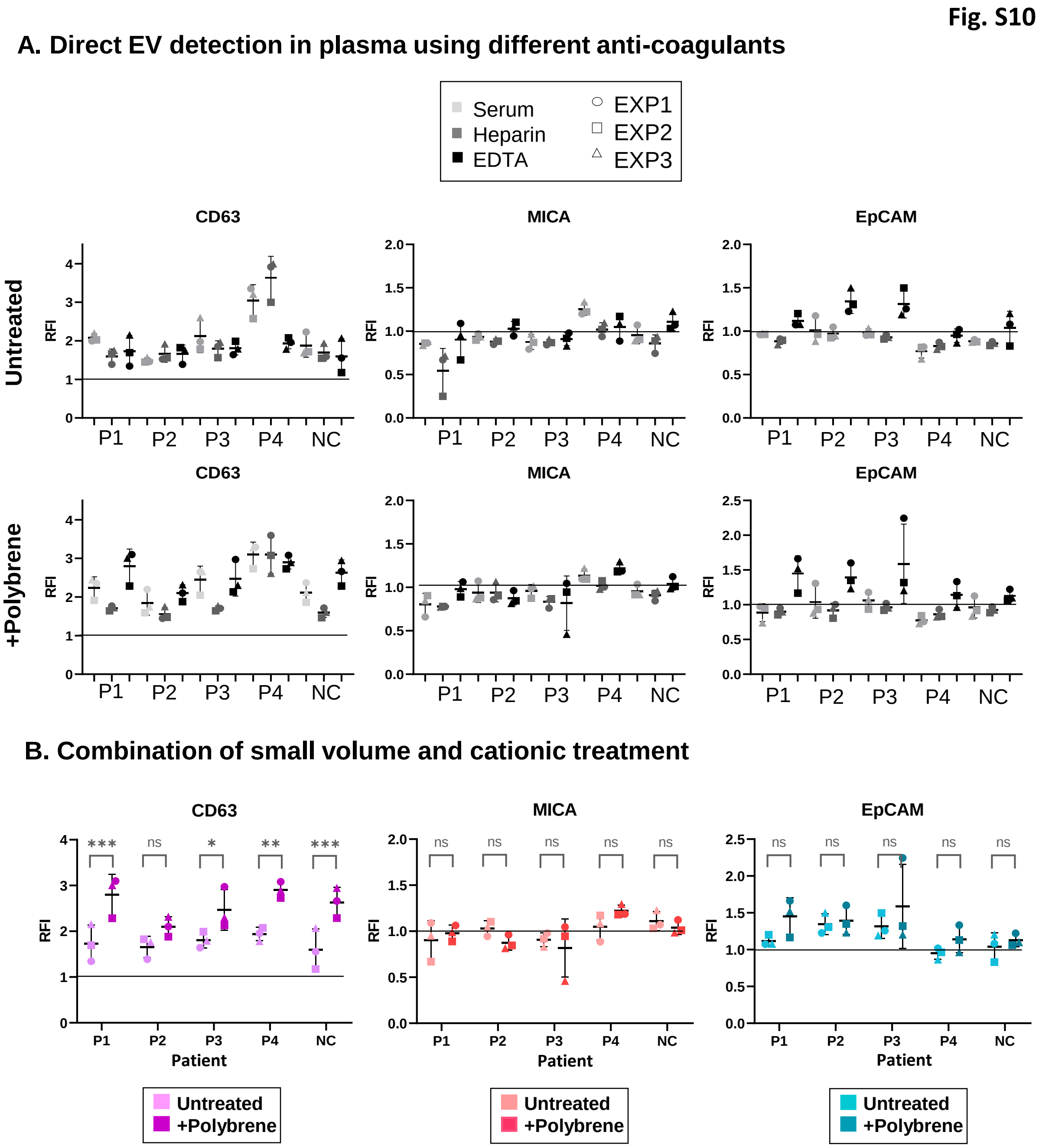

Direct EpCAM and MICA detection in cancer patient plasma can be improved by the combination of small volume and cationic treatment. **A. Direct EpCAM and MICA detection in cancer patient plasma.** 12 µl of PBS 1% casein containing 3000 beads conjugated with anti-CD63, anti-EpCAM or anti-MICA, as indicated, were incubated for 16 h with 12 µl of either serum (obtained in EDTA-tubes or heparin tubes) or plasma from each patient (cancer P1-P4 and non-cancer NC patients). The final volume of the assay was 26.5 µl, and two conditions were tested: either untreated EVs (in PBS 1% casein) (upper row) or treated with Polybrene at 8 µg/mL (lower row). EVs captured in each assay were detected with anti-CD81-PE. The signal obtained from incubation of plasma with IgG isotype control-coated beads was used to calculate the Relative Fluorescence Intensity (RFI). Mean and Standard Deviation from three independent experiments are represented. Statistical analysis was performed by a multiple t-test correcting for multiple comparisons by the Holm Sidack method ($p < 0.05$). * $p < 0.05$, ** $p < 0.01$, *** $p < 0.001$, **** $p < 0.0001$). Patient samples were obtained at Clínica Universidad de Navarra. **B.** 12 µl of PBS-casein containing 3000 beads conjugated with anti-CD63, anti-EpCAM or anti-MICA, as indicated, were incubated for 16 h with 12 µl of EDTA-plasma from each patient (cancer P1-P4 and non-cancer NC patients) either treated with Polybrene at 8 µg/mL or untreated with polymer. The final volume of the assay was 26.5 µl. Captured EVs were detected with anti-CD81-PE. The signal obtained from incubation of plasma with IgG isotype control-coated beads was used to calculate the Relative Fluorescence Intensity (RFI). Mean and Standard Deviation from three independent experiments are represented. Statistical analysis was performed by a multiple t-test correcting for multiple comparisons by the Holm Sidack method ($p < 0.05$). * $p < 0.05$, ** $p < 0.01$, *** $p < 0.001$, **** $p < 0.0001$). Patient samples were obtained at Clínica Universidad de Navarra.

Fig. S11

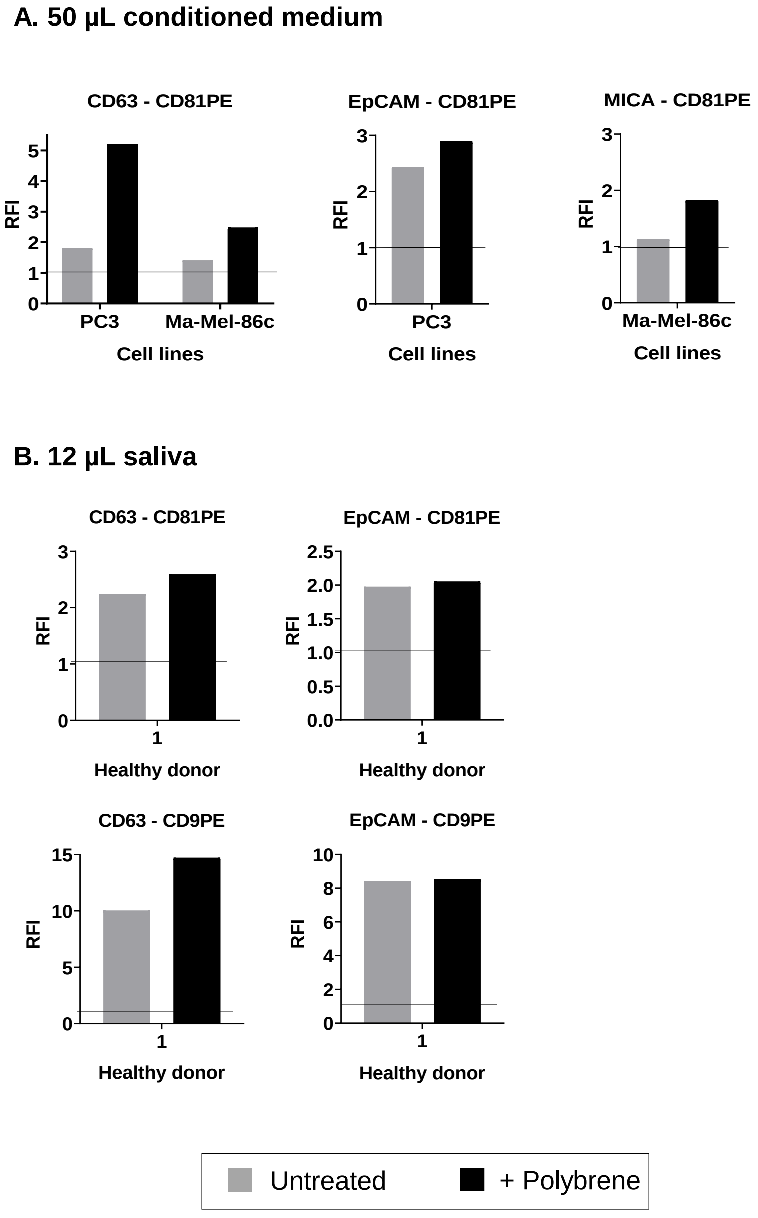

Tetraspanins and EpCAM can be detected directly in minimal volumes of different biological fluids by flow cytometry. Polybrene can enhance low signals. PBS 1% casein containing 3000 beads conjugated either with anti-CD63, anti-MICA or anti-EpCAM (as indicated) were incubated for 16 h with the indicated volumes of conditioned medium (A), or saliva from a healthy donor (B), either untreated (PBS 1% casein) or treated with polybrene at 8 µg/mL. The final volume of the assay was 100 µl in A, and 26.5 µl in B. EVs captured in each assay were detected with anti-CD81-PE or anti-CD9-PE as indicated. Isotype-PE was used as a negative control to calculate the RFI: Relative Fluorescence Intensity. Samples were centrifuged once 10 min at 200 x g before analysis, except saliva which was centrifuged twice.
